# Exosomes in the tumor microenvironment of cholangiocarcinoma: current status and future perspectives

**DOI:** 10.1186/s12967-022-03294-x

**Published:** 2022-03-07

**Authors:** Kai Zhao, Xiangyu Li, Yuanxin Shi, Yun Lu, Peng Qiu, Zhengdong Deng, Wei Yao, Jianming Wang

**Affiliations:** 1 Department of Biliary and Pancreatic Surgery/Cancer Research Center Affiliated Tongji Hospital, Tongji Medical College, Huazhong University of Science and Technology, Wuhan, 430030 Hubei China; 2 Department of Oncology Affiliated Tongji Hospital, Tongji Medical College, Huazhong University of Science and Technology, Wuhan, 430030 Hubei China; 3 Affiliated Tianyou Hospital, Wuhan University of Science and Technology, Wuhan, 430064 China

**Keywords:** Exosomes, Cholangiocarcinoma, Tumor microenvironment, Liquid biopsy, Biomarker, Targeted therapy

## Abstract

Cholangiocarcinoma (CCA) refers to an aggressive malignancy with a high fatality rate and poor prognosis. Globally, the morbidity of CCA is increasing for the past few decades, which has progressed into a disease that gravely endangers human health. Exosomes belong to a class of extracellular vesicles (EVs) with diameters ranging from 40 to 150 nm that can be discharged by all living cells. As communication messengers of the intercellular network, exosomes carry a diverse range of cargoes such as proteins, nucleic acids, lipids, and metabolic substances, which are capable of conveying biological information across different cell types to mediate various physiological activities or pathological changes. Increasing studies have demonstrated that exosomes in the tumor microenvironment participate in regulating tumorigenesis and progression via multiple approaches in the tumor microenvironment. Here, we reviewed the current research progress of exosomes in the context of cancer and particularly highlighted their functions in modulating the development of CCA. Furthermore, the potential values of exosomes as diagnostic and therapeutic targets in CCA were overviewed as well.

## Background

CCA remains a highly lethal malignancy of the biliary system. It can be classified into three major groups based on their lesion locations: intrahepatic, perihilar, and distal CCA. Globally, the proportion of CCA is second only to hepatocellular carcinoma (HCC) among all primary liver tumors, accounting for roughly 15%, and accounts for 3% of all gastrointestinal malignant tumors [1, 2]. Despite advancements advances in CCA cognition, diagnosis, and treatment for the past few years, due to its high malignant degree, strong invasiveness, and the occult in initial, most patients are first diagnosed already in the late-stage that severely constrains therapeutic options. Although curative surgery is a preferred selection for some early-stage patients, the fact of tumor recurrence or metastasis after resection remains frustrating. Meanwhile, on account of the high heterogeneity of CCAs, the systemic therapeutics bring little effect for advanced patients who are not possible for radical surgery, leading to a very poor prognosis that only 7%-20% of patients can reach the five-year survival [3, 4]. Nevertheless, with the advancements in genetic profiling of CCAs, emerging treatments such as targeted or immunological therapeutics may be able to assist patients with this deadly cancer to get better outcomes. Considering the current situation of CCAs, exploring new diagnostic and therapeutic strategies remains a matter of priority.

EVs are enveloped by a lipid bilayer that can be discharged by numerous cell sorts. According to the size and formation pathway of vesicles, it can be classified into two major subsets approximately, called ectosomes and exosomes [5]. The former are vesicles with a diameter of 50 nm ~ 1 μm via plasma membrane budding outward directly, while exosomes are EVs ranging from 40 ~ 150 nm in diameter generated in the opposite way, which involves plasma membrane invagination and endosomal formation [5]. Exosomes contain multiple substances and are broadly distributed in different body fluids like plasma, urine, bile, and cerebrospinal fluid (CSF), which play important roles in a variety of normal or abnormal biological behaviors [6–8]. Recently, researches about cancer exosomes have received tremendous attention. Intercellular communication in the microenvironment plays a significant role in regulating tumor development, where exosomes are key messengers that mediate this cell-to-cell communication [5, 9]. Previous researches have illustrated that exosomes participate in tumorigenesis or metastasis in multiple ways, their potential usages in cancer diagnosis and prognosis have also been deeply explored [9]. Although certain studies have reviewed the roles of EVs in the progression of CCA [10], a more comprehensive summarization of exosomes in CCA remains insufficient up to now.

In this article, we systematically summarized the research status of exosomes in the tumor fields. Based on the existing researches of exosomes in CCA, we specifically emphasized their significant roles in regulating tumor development and potential values in diagnosis and treatment.

## Research status of exosomes

### Biogenesis, secretion and internalization

As a type of EVs, the synthesis progress of exosomes involves three major phases: 1) plasma membrane invagination and early endosomes formation. 2) intraluminal vesicles (ILVs) and intracellular multivesicular bodies (MVBs) generation. 3) the fusion of MVBs and plasma membrane leads to exosomes secretion [9]. Generally, the biogenesis of MVBs mainly depends on the following two pathways: endosomal sorting complexes required for transport (ESCRT)-dependent or ESCRT-independent mechanisms, and the former is the most classic pathway [6]. Once mature, MVBs can integrate with autophagosomes then degrade through the lysosomal pathway or secrete into extracellular space as exosomes by fusing with the plasma membrane [11]. In this biogenesis and secretion process, other components such as tumor susceptibility gene 101 (TSG101), Rab family of GTPases (like Rab27A and Rab27B), soluble N-ethylmaleimide-sensitive factor attachment protein receptor (SNARE) complexes, apoptosis-linked gene 2-interacting protein X (Alix), ceramide, tetraspanins (CD63, CD9, CD81), and phospholipids are also getting involved [5, 12, 13].

**Fig. 1:**
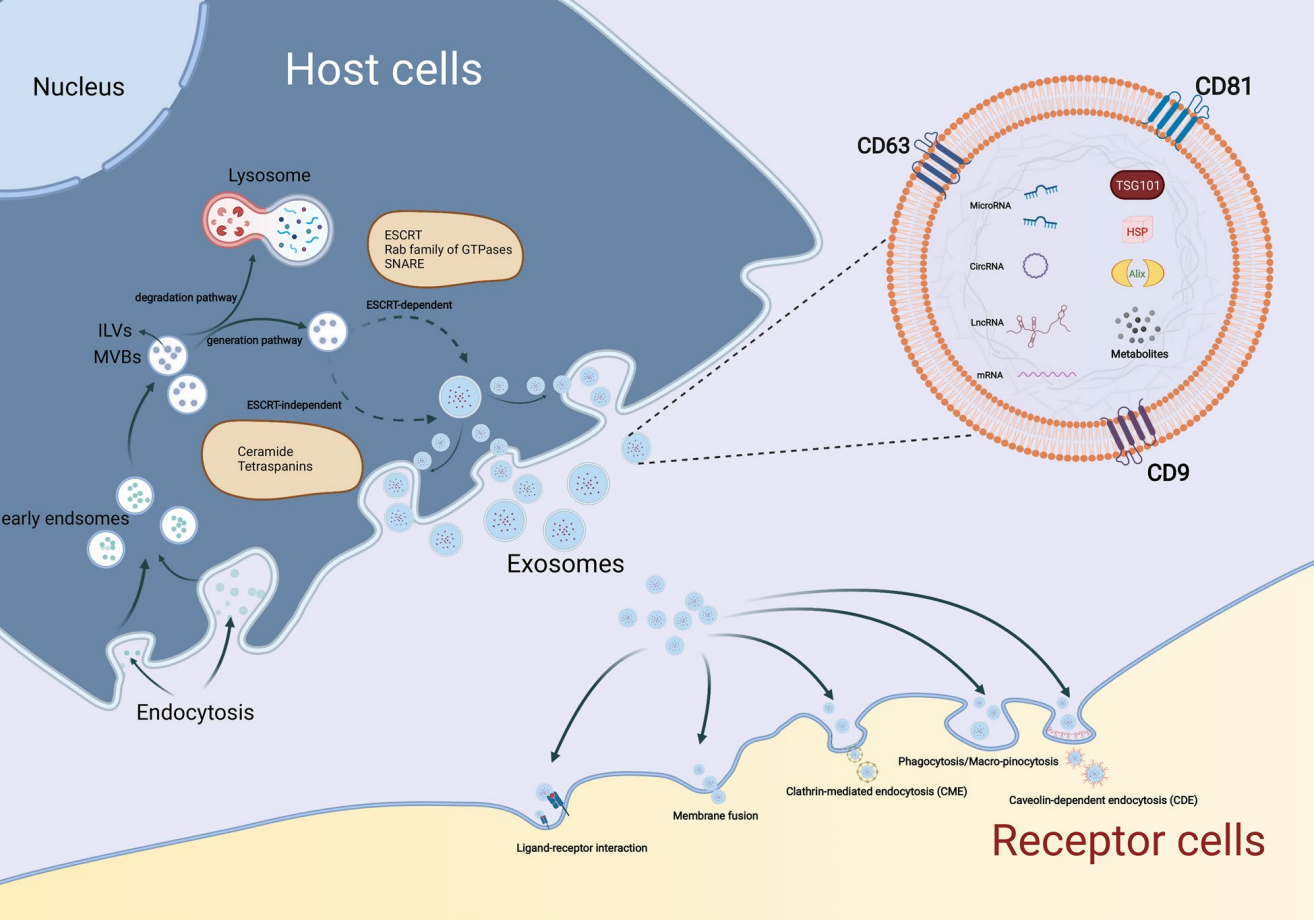
Biogenesis, secretion, and internalization of exosomes. The formation of exosomes initially depends on the invagination of the plasma membrane, followed by the generation of ILVs and MVBs. Once mature, MVBs can fuse with lysosomes and be degraded, or integrate with the plasma membrane and finally get released, i.e., exosomes. During this process of synthesis and secretion, ESCRT-dependent and ESCRT-independent mechanisms are two common approaches, other components like the Rab family of GTPases, SNARE, ceramide, and tetraspanins are also involved. Exosomes can be uptake by receptor cells to perform specific functions through various mechanisms, such as phagocytosis, macro-pinocytosis, ligand-receptor interaction, CME, and CDE. As plasma membrane-derived vesicles with lipid bilayer structure, exosomes carry a variety of components, including RNAs (mRNA, MiRNA, LncRNA, and CircRNA), proteins (TSG101, Alix, HSP, CD9, CD63, and CD81) and metabolites, etc.

Considering the difference in the origin and microenvironment, exosomes have a strong heterogeneity, which is mainly reflected in the regulation of the target cell functions [5]. Once exosomes are secreted by the host cells, they can be absorbed by target cells through various approaches like endocytosis, plasma membrane integration, and specific protein interactions [14]. Among these internalization ways, endocytosis is the most widely studied pattern. According to the characteristics of components involved in endocytosis, several subtypes are broadly divided up, including phagocytosis, macro-pinocytosis, clathrin-mediated endocytosis (CME), and caveolin-dependent endocytosis (CDE), as well as lipid raft-mediated internalization [15]. Moreover, several proteins such as tetraspanins, integrins, proteoglycans, and lectins, also participate in the internalization of exosomes by the unique ligand-receptor interactions[9]. However, on account of the heterogeneity of exosomes, whether or not exosomes uptake is specific remains controversial(15). Therefore, it is essential to further investigate the detailed routes of exosomes uptake (Fig. 1).

## Isolation and identification

Currently, frequently-used isolation strategies include centrifugation (differential or density gradient centrifugation), particle size separation, size-exclusion chromatography, microfluidic technique, and immunoaffinity capture [16]. Until now, the most common method is still differential centrifugation due to its high exosome yields and relatively cheap price. However, it also has some deficiencies like complicated procedures, low separation efficiency, and susceptibility to contamination by soluble substances in cell culture medium or other body fluids [17]. Other isolation methods like size-exclusion chromatography, with a relatively high yield but difficult to achieve mass production, and immunoaffinity capture, advanced in specific separation yet costly with low yields [16, 17]. So far there is not a standardized method that can achieve both economic and high purity at the same time. Therefore, the exploration of better purification methods remains a major challenge in the exosome-related fields.

In terms of identification, the International Society of Extracellular Vesicles proposed to identify exosomes mainly from the following three aspects: 1) Exosomal morphology identification, 2) Exosomal size detection, 3) Exosomal biomarkers identification [18, 19]. Among them, transmission electron microscopy (TEM), cryo-electron microscopy (Cryo-EM), and atomic force microscopy (AFM) are the most direct methods for visual observation of exosomes [20]. Real-time nanoparticle tracking technology based on the principle of Brownian motion can be used to obtain the size distribution of exosomes [21]. In addition, enzyme-linked immunosorbent assay (ELISA), flow cytometry (FCM), and western blotting (WB) are available means to detect the specific proteins or other markers expressed on exosomes [20, 22]. Reportedly, several transmembrane proteins like CD9, CD63, and CD81 are considered to be representative hallmarks, however, a recent study suggested that compared with other tetraspanins, CD63 is the unique biomarker, while CD9 and CD81 are not specific for exosomes [23]. Moreover, other components related to the formation of exosomes such as Alix, TSG101, and Heat shock proteins (HSP) can also serve as classical hallmarks [5].

## The roles of exosomes in malignancies

Since exosomes have played an essential role in multiple pathological changes through mediating intercellular communication, it has also received enormous concerns in the cancer area over the past few years [9]. Related studies have pointed out that cancer-cell-derived exosomes can modulate tumor progression through a variety of mechanisms [9, 24]. Besides, as mentioned above, exosomes contain complex cargoes that are widespread in various body fluids, which also partly represent the heterogeneity of their parental cells, making them available for cancer diagnosis and prognostic by serving as novel biomarkers [25]. Moreover, recent studies have focused more on the tumor microenvironment (TME), where the signal interaction mediated by exosomes also makes a difference in tumor development [5, 26].

Exosomes induce or accelerate tumorigenesis. Exosomes secreted by HCC cancer cells promoted tumorigenesis through the Hedgehog pathway [27]. Mirna-224-5p-enriched exosomes secreted by non-small cell lung cancer (NSCLC) cells accelerated neoplasia by directly binding with the androgen receptor (AR) [28]. On the contrary, exosomes distributed in the plasma of patients with medulloblastoma inhibited tumorigenesis by targeting FOXP4 (forkhead box protein 4) and EZH2 (enhancer of zeste 2 polycomb repressive complex 2 subunit) directly through their miRNA cargoes [29].

Exosomes have also been shown to be involved in tumor angiogenesis, which is a critical step in tumor progression. Exosomes loaded with miR-205 secreted by tumor cells induced angiogenesis via the PTEN(phosphatase and tensin homolog)/AKT pathway in Ovarian cancer [30]. Exosomal miR-25-3p derived from Colorectal cancer (CRC) cells could be absorbed by endothelial cells to facilitate, angiogenesis and increasing vascular permeability via targeting KLF2 (kruppel like factor 2) and KLF4 (kruppel like factor 4). Moreover, both in vitro study and clinical data suggested that exosomal miR-25-3p also related to the formation of pre-metastatic niche, making it a promising sign for CRC metastasis [31]. In the context of cancer, soluble E-cadherin (sE-cad)-enriched exosomes were potent stimulators of angiogenesis and might may relate to the formation of malignant ascites and widespread peritoneal metastasis in ovarian cancer patients [32].

TME consists of a group of cellular and noncellular components, including fibroblasts, immune cells such as macrophages, neutrophils, and lymphocytes, as well as cytokines, blood vessels, and extracellular matrix, etc [26]. And the crosstalk among different cell types mediated by exosomes has proven to be strongly related to tumor progression and therapeutic response. For one perspective, cancer-cell-derived exosomes regulate the function of stromal cells in the microenvironment. For example, exosomes from HCC cells induced the activation of cancer-associated fibroblasts (CAFs) activation to promote lung metastasis through their miRNA cargoes [33]. Exosomes secreted by epithelial ovarian cancer (EOC) cells under hypoxic conditions could regulate the macrophage polarization by transferring their miRNAs (miR-21-3p, miR-125b-5p, miR-181d-5p) to promote tumor proliferation and metastasis [34]. From another perspective, exosomes originated from other infiltrating cells in the TME can also modulate the biological behavior of cancer cells. Exosomal miR-34a-5p could transfer from CAFs to cancer cells, subsequently induced epithelial-mesenchymal transition (EMT) via the AKT/GSK-3β(glycogen synthase kinase 3 beta)/β-catenin pathway in oral squamous cell carcinoma (OSCC) [35]. Exosomes derived from M2 macrophage mediated an intercellular transfer of the integrin α_M_β_2_ and promoted HCC metastasis through activating the MMP9 (matrix metalloproteinase 9) signaling pathway [36]. Moreover, the functions of exosomes in tumor immunity have also been explored to some degree. Hypoxia-induced tumor exosomes were abundant in chemokines and cytokines like CSF-1(colony stimulating factor 1), MCP-1(monocyte chemoattractant protein-1), and TGFβ (transforming growth factor beta), which can modify the host immune microenvironment and enhance tumor progression via influencing the macrophage recruitment and polarization [37]. Circ-UHRF1 (ubiquitin-like with PHD and ring finger domain 1), existed in plasma exosomes secreted by HCC patients, which leading to immunosuppression by inhibiting NKs (natural killer cells) activity via circUHRF1/miR-449c-5p/TIM-3(T cell immunoglobulin domain and mucin domain 3) axis [38]. While such exosome-mediated signal transmission can exert antitumor effects under certain circumstances. For example, exosomes derived from DCs (dendritic cells) were reported that might become a novel vaccine applied in tumor immunotherapy. Exosomes secreted by α-fetoprotein (AFP)-positive DCs could effectively improve the immune microenvironment of mice models with HCC, making it a hopeful new strategy for immunotherapy of HCC [39]. In addition to the components contained in exosomes, their external molecules also participate in tumor immunoregulation. PD-L1(programmed death 1 ligand), known as a natural ligand for PD-1 (programmed death 1), can suppress the immunocompetence of T cells, B cells, and monocytes by directly binding with PD-1 on their surface to promote tumor immune escape [40]. It has been demonstrated that anti-PD-1/PD-L1 therapeutics has achieved a great success in multiple cancers, including metastatic melanoma, NSCLC, glioblastoma, and colon cancer, while the problem of drug resistance largely limits their clinical application [41]. Recent researches have indicated that exosomes derived from cancer cells also express PD-L1. PD-L1^+^ exosomes can impair immune functions and promote tumor growth in the similar way as above, which may also result in a low response to anti-PD-L1 therapy. Therefore, targeting the PD-L1 expressed on exosomes is expected to improve the present situation of cancer immunotherapy [40, 42].

Palliative treatments like radiotherapy, chemotherapy, and targeted therapy are recommended choices for the later period tumor patients, while it is also disappointing because tumors are prone to become tolerant of these chemotherapeutic agents or antibodies. And increasing evidence supports that exosomes can facilitate this resistance by mediating intercellular communication. LncRNA SNHG7 (small nucleolar RNA host gene 7) transmitted between cancer cells in exosomes enhanced docetaxel resistance in lung adenocarcinoma (LUAD) by inducing autophagy and regulating the macrophage polarization [43]. And tumor-associated macrophages (TAMs) derived exosomes were capable of inducing drug resistance in multiple cancers through their miRNA or lncRNA cargoes as well [44–47]. Moreover, miRNA-522, abundant in exosomes released from CAFs, could promote acquired chemotherapy resistance and inhibit ferroptosis, thus supporting tumor progression of gastric cancer [48]. In addition, other researchers have also noted that exosomes play a unique role in improving drug resistance. For example, miR-567-enriched exosomes reversed trastuzumab resistance via suppressing autophagy by targeting ATG5(autophagy related 5), promising to serve as a potential therapeutic target for breast cancer patients [49].

As for the value of exosomes in tumor diagnosis and prognosis, there is a lot of researches to back it up. Previous studies mainly focused on its nucleic acids cargoes, especially small RNA molecules such as classical oncogenic miR-21, miR-155, and anti-oncogenic miRNAs like miR-146a and miR-34a, which are differentially expressed between tumor cells and non-tumor cells, enabling them to diagnose multiple cancers at an early-stage, like pancreas, colorectum, liver, and breast cancers [50]. Besides miRNAs, exosomal proteins also have clinical significance [51]. Glypican-1 (GPC1)-positive exosomes could be used as an early diagnosis tool for patients with pancreatic cancers, which also performed better in prognosis prediction compared with CA19-9 [52]. By constructing mouse liver damage models and the proteomic analysis of their urine exosomes, twenty-eight novel proteins were identified and four of them are promising were expected to be used as non-invasive indicators in hepatic disease [53]. Based on the evidence of these researches, the combination of multiple components involved in exosomes may help enhance the specificity and sensitivity of cancer diagnosis, while further research is still needed.

To sum up, exosomes are closely related to tumor progression, and it has been well explored in a wide range of digestive malignancies. However, relevant studies in CCA are still insufficient, and existing researches have not been systematically described either. Here, the current research progress of exosomes in CCA will be comprehensively introduced in the next part.

## Roles of exosomes in CCA

### Exosomes in CCA progression

As the most well-studied component of exosomes, the critical roles of ncRNAs in CCA have been extensively reported as well. Several dysregulated exosomal miRNAs have been detected between CCA cells and normal biliary epithelial cells through miRNA profiling analysis. Among these differentially expressed miRNAs, miR-205-5p, miR-200c-3p and miR-200b-3p, were significantly abundant in exosomes, while miR-34c-5p and miR-199 clusters were down-regulated distinctly. Subsequently, KEGG (kyoto encyclopedia of genes and genomes) enrichment analysis of target genes predicted by differentially expressed miRNAs suggested that dysregulated miRNAs were closely related to multiple cancer-associated pathways, and down-regulating the expression of miR-205-5p effectively suppressed the invasion and metastasis of CCA cells in vitro, indicating that it might take an essential part in CCA progression [54]. And in the CCA microenvironment, EVs derived from cancer cells could induce bone marrow mesenchymal stem cells (BMSCs) to differentiate into fibroblasts, accompanied by a significant up-regulation in the myofibroblast markers like α-SMA (alpha-smooth muscle actin), FAP (fibroblast activation protein alpha), and vimentin. Such differentiation enhanced the migratory capability of BMSCs and contributed to tumor extracellular matrix formation, facilitating the development of CCA ultimately. As a response, some soluble mediators such as IL-6 (interleukin- 6) could be selectively released from BMSCs as well, and in turn, enhancing the proliferation capacity of tumor cells by stimulating the STAT3 (signal transducer and activator of transcription 3) signaling pathway [55]. Another study focused on the interaction between CCA cells and fibroblasts observed that down-regulating the miR-34c in exosomes derived from CCA cells could mediate the activation of CAFs to promote cancer development [56].

Interestingly, certain miRNAs transferred in the form of exosomes can also act as tumor suppressors. In the co-culture system of CCA cell line HuCCT1 and hepatic stellate cell line LX2, several markedly down-regulated miRNAs in LX2 cells were identified, and among them, miR-195 received more attention. Further functional experiments showed that the capacities of growth and invasion of CCA cells decreased significantly after up-regulating the miR-195 in LX2 cells, indicating that miR-195 might act as a tumor suppressor in this cell culture model. Subsequent studies on the mechanism demonstrated that miR-195 was loaded in EVs secreted by LX2 cells, and performed this anti-tumor effect by directly transferring to CCA cells [57]. Similarly, intercellular transmission of miR-30e in EVs can also inhibit EMT of receptor cells via targeting Snail and then suppress invasion and metastasis of CCA [58].

Besides miRNAs, other exosomal ncRNAs like circRNA and lncRNA have been reported in CCA as well. Circ-0000284 was markedly up-regulated in CCA cells compared to normal bile duct cells, promoted CCA development as a competitive endogenous RNA via miR-637/LY6E (lymphocyte antigen 6 family member E) pathway. The study also pointed out that circ-0000284 transmission via exosomes could induce a malignant transformation of normal cells adjacent to cancer [59]. Circ-CCAC1 (Cholangiocarcinoma-associated circular RNA 1) facilitated CCA growth and migration via sponging miR-514a-5p to elevate the expression of YY1 (Yin Yang 1) and CAMLG (calcium modulating ligand). And a high level of circ-CCAC1 was detected CCA-derived EVs, these EVs could be transmitted to endothelial cells to disrupt the continuity of vascular endothelial barrier and stimulate the formation of new blood vessels, promoting tumor growth and metastasis in turn [60].

In addition to nucleic acid molecules, the protein components contained in exosomes are also related to the malignant progression of CCA. For example, FZD 10 (frizzled class receptor 10) proteins, related to the Wnt signaling pathway, were detected in CCA-derived exosomes that could promote cell proliferation and might be involved in mediating the cancer reactivation and distant metastasis [61]. Several common cancer-related proteins, like integrin α/β, lactadherin, and vitronectin, were identified in CCA-derived exosomes, could induce invasion and migration of cholangiocytes by up-regulating the expression of β-catenin [62].

As mentioned above, exosomes also participate in regulating tumor progression by modulating the immune microenvironment. And in CCA, Chen et al. have observed that cancer-related exosomes could reduce the population of cytokine-induced killer cells (CIKs), leading to a secretory reduction of TNF-α (tumor necrosis factor-α) and perforin, thus inhibiting anti-tumor activity and promoting tumor immune escape ultimately [63].

**Fig. 2:**
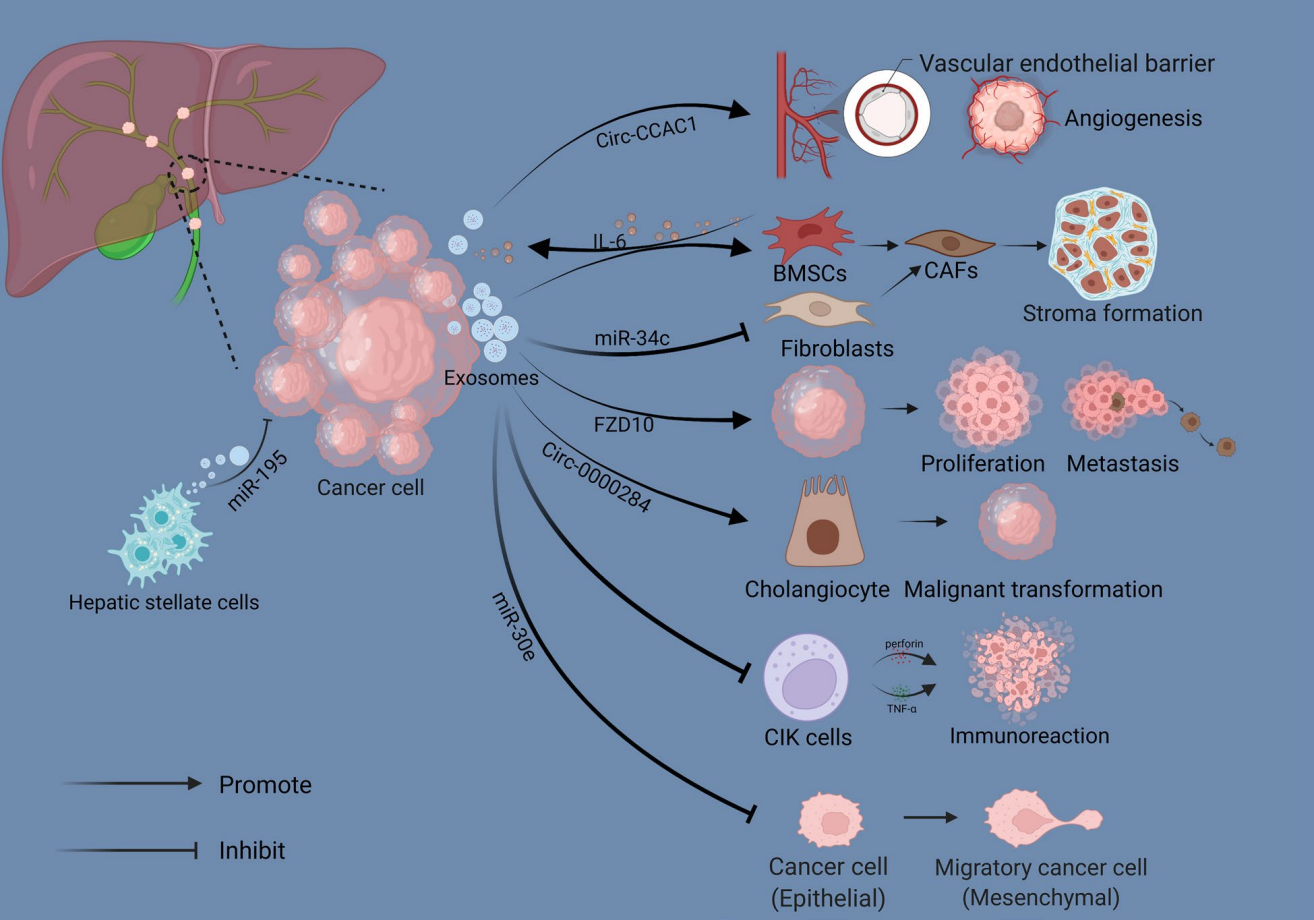
The functional network of exosomes in the CCA microenvironment. Exosomes secreted by cancer cells facilitate tumor angiogenesis by transferring Circ-CCAC1 to endothelial cells. Cancer cell-derived exosomes induce BMSCs to differentiate into CAFs to promote tumor stroma formation, and normal fibroblasts can also be activated through their miRNA cargoes, like miR-34c. Other RNAs cargoes, such as Circ-0000284 can promote tumor development by inducing a malignant transformation of normal cholangiocytes, while miR-30e-enriched exosomes inhibit tumor progression via suppressing EMT. Similarly, exosomes derived from Hepatic stellate cells exert an anti-tumor effect by transferring miR-195. Certain protein components in exosomes can also promote tumor proliferation and metastasis, like FZD10. CIKs absorb exosomes to promote tumor immune escape by reducing the secretion of TNF-α and perforin

In conclusion, these studies have confirmed that exosomes are closely related to the progression of CCA, providing a new perspective for understanding the regulatory mechanisms of tumor development (Fig. 2).

## Exosomes in CCA diagnosis

Due to the stealthiness and heterogeneity of CCA, traditional image logical examination or laboratory test cannot achieve early diagnosis or dig out characteristics that reflect tumorigenesis. Recently, an emerging method of diagnosis called “liquid biopsy” has attracted more attention that may make up for the deficiency of traditional detection methods in cancer diagnosis. Several explorations aimed to develop the exosomes as objects for liquid biopsy have also been conducted in CCA.

Several differentially expressed exosomal RNAs have been detected in serum or urine of CCA patients, compared with primary sclerosing cholangitis (PSC), ulcerative colitis (UC), and healthy controls. Among them, five serum exosomal mRNAs including CMIP (c-Maf inducing protein), GAD1 (glutamate decarboxylase 1), NDKP1 (nucleoside diphosphate kinase 1), CDS1 (CDP-diacylglycerol synthase 1), and CKS1B (cyclin-dependent kinase regulatory subunit 1), as well as a cluster of urine exosomal mRNAs consisted of UBE2C (ubiquitin-conjugating enzyme E2C) and SERPINB1 (serine protease inhibitor B1), showed an excellent performance in diagnostic efficiency. Particularly, uniting CMIP, NDKP1, and CKS1B performed a better diagnostic power. Moreover, exosomal ncRNAs [miR-604, miR-551b, and lncRNA MALAT1(metastasis associated lung adenocarcinoma transcript 1)] in the serum of CCA patients also contributed to the diagnosis [64]. Similarly, in another study of serum exosomes, twenty exosomal miRNAs were identified and five of them had a significant differential expression. Further assessment of diagnostic value using these dysregulated miRNAs showed that miR-200 family members (miR-141-3p, miR-200a-3p, miR-200b-3p, and miR-200c-3p) performed better than CA19-9. Particularly miR-200c-3p, which is positively related to tumor stage, was expected to be a novel serum indicator for CCA progression [65]. Conversely, certain miRNAs down-regulated in exosomes also possessed diagnostic values, such as miR-214-3p and miR-199 family members [54]. And the latest research noted that Cripto-1 [also named teratocarcinoma-derived growth factor 1 (TDGF-1)], largely existed in exosomes isolated from the serum of perihilar cholangiocarcinoma (PHCCA) patients versus cholangitis patients and normal controls, performed a better diagnostic capacity than CEA and CA19-9 (AUC: 0.82; Sensitivity: 79.1%; Specificity: 87.5%). Furthermore, Immunohistochemistry analysis using PHCCA tissues showed a negative correlation between Cripto-1 and E-cadherin, indicating that Cripto-1 was also related to EMT and may possess an independent prognostic significance in PHCCA [66].

Researches aimed at bile-derived exosomes have also identified several circulating exosomal lncRNAs, which are closely related to oncogenic signaling pathways (like p53 and RAS signaling pathways) that may be conducive to the diagnosis of CCA [67]. Another high-quality research has identified four miRNAs (miR-96-5p, miR-191-5p, miR-151a-5p, and miR-4732-3p) that enriched in exosomes isolated from blood samples of CCA patients, which are promising for early diagnosis of CCA, especially stage II patients [68].

In addition, some researchers have paid more attention to the proteins contained in CCA exosomes. By using proteomics approaches, Heat shock protein 90 (HSP90) was confirmed that differentially phosphorylated in invasive CCA cells and was expected to serve as an implement for indicating the metastatic of CCA [69]. Besides, proteomic analysis showed that Claudin-3 was enriched in human bile-derived exosomes, and might become a novel biomarker for CCA as well [70]. In comparison with serum exosomes isolated from normal individuals, several specific proteins were observed to be concentrated in exosomes of CCA patients. In detail, a combination of three of them, including AMPN (aminopeptidase N), VNN1 (pantetheinase), and PIGR (polymeric immunoglobulin receptor), performed better in diagnosis that might become alternative serum biomarkers in CCA. And in the same study, another three exosomal proteins: FIBG (fibrinogen gamma chain), A1AG1 (alpha1-acid glycoprotein 1), and S100A8 (S100 calcium binding protein A8), were expected to become an effective basis for differential diagnosis with PSC [71].

In summary, these researches have emphasized the potential value of exosomes in CCA diagnosis and prognosis, while large cohort validation is still needed in the future (Table 1).

**Table 1:** Exosomes as diagnostic and prognostic biomarkers in cholangiocarcinoma

Biomarker	Molecule type	Expression (compared to control group)	Location	Sample	Case group	Control group	Diagnosis/Prognosis	References
miR-604, miR-551b	miRNA	Up-regulated	Serum	12 CCA patients, 6 PSC patients, 8 UC patients, 9 Healthy controls	CCA patients (n = 12)	PSC patients (n = 6), UC patients (n = 8), Healthy individuals (n = 9)	Diagnosis	[53]
miR-141-3p, miR-200a-3p, miR-200b-3p, miR-200c-3p	miRNA	Up-regulated	Serum	36 CCA patients, 12 Healthy controls	CCA patients (n = 36)	Healthy controls (n = 12)	Diagnosis and prognosis	[54]
miR-199 family, miR-214-3p	miRNA	Down-regulated	CCA cell supernatant	human CCA cells, normal human cholangiocytes	human CCA cells	normal human cholangiocytes	Diagnosis and prognosis	[43]
miR-96-5p, miR-151a-5p, miR-191-5p, miR-4732-3p	miRNA	Up-regulated	Blood	45 CCA patients, 40 Healthy controls	CCA patients (n = 45)	Healthy controls (n = 40)	Diagnosis	[57]
LncRNA MALAT1	LncRNA	Up-regulated	Serum	12 CCA patients, 6 PSC patients, 8 UC patients, 9 Healthy controls	CCA patients (n = 12)	PSC patients (n = 6), UC patients (n = 8), Healthy individuals (n = 9)	Diagnosis	[53]
ENST00000588480.1/ENST00000517758.1	LncRNA	Up-regulated	Bile	35 CCA patients, 56 Biliary obstruction patients	CCA patients (n = 35)	Biliary obstruction patients (n = 56)	Diagnosis and prognosis	[56]
Cripto-1	mRNA	Up-regulated	Serum	115 PHCCA patients, 47 cholangitis patients, 65 Healthy controls	PHCCA patients (n = 115)	Cholangitis patients (n = 47), Healthy individuals (n = 65)	Diagnosis and prognosis	[55]
UBE2C, SERPINB1	mRNA	Up-regulated	Urine	23 CCA patients, 5 PSC patients, 12 UC patients, 5 Healthy controls	CCA patients (n = 23)	PSC patients (n = 5), UC patients (n = 12), Healthy individuals (n = 5)	Diagnosis	[53]
CMIP, GAD1, NDKP1, CDS1, CKS1B	mRNA	Up-regulated	Serum	12 CCA patients, 6 PSC patients, 8 UC patients, 9 Healthy controls	CCA patients (n = 12)	PSC patients (n = 6), UC patients (n = 8), Healthy individuals (n = 9)	Diagnosis	[53]
AMPN, VNN1, PIGR	Protein	Up-regulated	Serum	43 CCA patients, 32 Healthy controls	CCA patients (n = 43)	Healthy individuals (n = 32)	Diagnosis	[60]
FIBG, A1AG1, S100A8	Protein	Up-regulated	Serum	43 CCA patients, 30 PSC patients	CCA patients (n = 43)	PSC patients (n = 30)	Diagnosis	[60]
HSP90	Protein	Low phosphorylated	CCA cell supernatant	human CCA cell lines: KKU-M213(M213) and KKU-M213D5 (M213D5)	M213D5 cells (highly invasive)	M213 cells	Diagnosis and prognosis	[58]
Claudin-3	Protein	Up-regulated	Bile	10 CCA patients, 10 Choledocholithiasis patients	CCA patients (n = 10)	Choledocholithiasis patients (n = 10)	Diagnosis	[59]

*MALAT1* metastasis associated lung adenocarcinoma transcript 1, *Cripto-1* teratocarcinoma-derived growth factor 1 (TDGF-1), *UBE2C* ubiquitin-conjugating enzyme E2C, *SERPINB1* serine protease inhibitor B1, *CMIP* c-Maf inducing protein, *GAD1* glutamate decarboxylase 1, *NDKP1* nucleoside diphosphate kinase 1, *CDS1* CDP-diacylglycerol synthase 1, *CKS1B* cyclin-dependent kinase regulatory subunit 1, *AMPN* aminopeptidase N, *VNN1* pantetheinase, *PIGR* polymeric immunoglobulin receptor, *FIBG* fibrinogen gamma chain, *A1AG1* alpha1-acid glycoprotein 1, *S100A8* S100 calcium binding protein A8, *PSC* primary sclerosing cholangitis, *UC* ulcerative colitis, *PHCCA* perihilar cholangiocarcinoma

## Progression in adjuvant therapy of CCA and the potential application of exosomes

At present, for CCA patients at an advanced stage that cannot accept radical surgery, gemcitabine combined with cisplatin is the preferred recommended option. More than three months longer median survival time is observed in patients who received this combination compared to gemcitabine treatment alone (gemcitabine + cisplatin group: 11.7 months; gemcitabine: 8.1 months) [72]. In addition, Rachna et al. have also reported that treatment with gemcitabine-cisplatin plus nab-paclitaxel displayed a better survival benefit versus gemcitabine-cisplatin alone in a phase II clinical trial [73], promising to become a new first-line therapeutic regimen for CCA. However, due to the significant heterogeneity of CCA, chemotherapy-based systemic treatments often have limited efficacy [74]. In recent years, with the maturity of genomics approaches, the molecular pathological mechanism of cholangiocarcinoma has been revealed gradually, providing new possibilities for individualized and targeted therapies [75].

Studies have found that different genomic profiles are shown in three CCA subtypes. To be more specific, patients with iCCA show a high mutation frequency in IDH1/2, KRAS, BAP1, TP53, as well as FGFR fusions, while PRKACA, PRKACB, and ELF3 mutations are more likely to appear in pCCA/dCCA patients [76, 77]. According to these mutations and fusions, CCA can be further divided into different molecular subtypes and prognostic assessment becomes available on this basis. Furthermore, several targeted drugs aimed at these mutations have already entered clinical trials [75]. For example, infigratinib (NVP-BGJ398) and erdafitinib, known as pan-FGFR inhibitors, displayed an excellent anti-tumor capacity, with a positive therapeutic response and controllable security, observed from relevant phase I and phase II clinical data [78, 79]. Moreover, immunotherapeutic strategies represented by anti-PD-1/anti-PD-L1 monoclonal antibodies have shown a significant remission rate in multiple human malignancies [80]. In advanced biliary tract cancers, several clinical trials have also reported that some progress has been made in patients who received pembrolizumab therapeutic (an immune checkpoint inhibitor targeting PD-1), with an objective response rate varying from 5.8% ~ 13% and a median progression-free survival of about 2 months [81]. However, relevant clinical exploration is still insufficient and unsatisfactory in CCA.

Exosomes are lipid bilayer-enveloped extracellular structures that can protect their cargoes from degradation. And most importantly, exosomes secreted by different cells can partly represent the heterogeneity of their parental cells, conferring them excellent biocompatibility compared to other drug delivery vehicles like liposomes or lipid-based nanoparticles. Therefore, it seems that exosomes are more suitable as drug carriers for cancer treatment [5]. Compared to direct therapy, exosomes as drug carriers perform better uptake efficiency and lower toxicity. Engineering exosome content to transport therapeutic nucleic acids, proteins, or drugs directly to cancer cells shows an exciting potential in cancer treatment [82]. For example, miR-122, which acted as an anti-tumor miRNA in HCC, could inhibit cancer development via suppressing EMT and angiogenesis and enhancing the chemosensitivity [83]. It was reported that adipose tissue-derived mesenchymal stem cells (AMSCs) derived exosomes could effectively transfer miR-122 to HCC cells, thereby increasing HCC chemosensitivity by modulating the expression of downstream molecules [84]. Similarly, transferring miR-199a-enriched exosomes from modified AMSCs to HCC cells enhanced the sensitivity of doxorubicin treatment by inhibiting the mTOR (mechanistic target of rapamycin kinase) signaling pathway [85]. Moreover, patients with pancreatic ductal adenocarcinoma are susceptible to becoming chemo-resistant, leading to a dismal therapeutic response and a poor prognosis. To improve this current status of treatment, Zhou et al. have loaded paclitaxel and gemcitabine monophosphate (an intermediate product of gemcitabine metabolism) into BMSCs-derived exosomes which can be absorbed by tumor cells. Further experiments showed an excellent penetration based on this Exo delivery platform, contributing to a favorable anti-tumor efficacy and relatively mild systemic toxicity [86]. These findings have opened up the way for exosomes to be used in tumor-targeted therapy.

Nevertheless, the application of exosomes as therapeutic targets in CCA is still in infancy, the current research focus is still inclined to their roles in tumor diagnosis and development, as we mentioned before. As for their therapeutic potential, existing studies have found that transferring several tumor-suppressive miRNAs (miR-195 and miR-30e) through exosomes can effectively inhibit CCA development [57, 58]. Vaccination of hamsters combined with Opisthorchis viverrini EVs and their surface recombinant tetraspanins has been demonstrated to reduce the burden of infection through inducing antibody reactions, exerting a protective role to avoid the appearance of CCA [87]. A relevant study reported that methotrexate-equipped EVs derived from cancer cells could effectively relieve malignant biliary obstruction by inducing pyroptotic cell death in patients with CCA [88]. Additionally, the latest research focused on the TME of CCA also brings forceful support for EV-based therapeutics. Exosomal miR-183-5p derived from cancer cells suppressed immune responses and promoted iCCA progression through up-regulating the expression of PD-L1 in macrophages via miR-183-5p/PTEN/AKT axis, promising to be a novel target for overcoming therapeutic tolerance of immune checkpoint inhibitors in iCCA [89]. Moreover, circ-0020256, produced by TAMs and loaded in exosomes, significantly facilitated the proliferation and metastasis of CCA cells, cutting off the cross-talk mediated by exosomal circ-0020256 between CCA cells and TAMs might be a hopefully therapeutic strategy [90]. All these findings provide strong evidence to support the application of exosomes in the treatment of CCA. However, mainly of them are based only on animal experiments, meaning that there is still a long way from clinical trials.

## Conclusions and perspectives

A growing number of studies support the thesis that the microenvironment plays a significant role in the development of multiple human cancers. Given that exosomes are key components of the microenvironment and act as messengers of intercellular communication, relevant studies have become hot topics of cancer research.

Exosomes can be separated from various body fluids, and certain RNAs, proteins, or metabolites involved in them have advantages in stability and abundance, giving them unique advantages in tumor diagnosis and prognosis. Moreover, since exosomes have excellent histocompatibility and can partly reflect the heterogeneity of their parental cells, modification of exosomes for cancer treatment also shows great potential. However, there are still many problems to be solved in the clinical application of exosomes. First, current exosome isolation and purification techniques need to be further optimized to achieve better efficiency and convenience. Next, the molecular mechanisms of exosomes in CCA malignant progression remains unclear, hence the exploration of internal details remains needed to develop potential therapeutic targets. Last but not least, the artificial synthetic exosome techniques are immature at the present stage, related clinical trials are also inadequate, thus developing exosomes as drug carriers in cancer treatment still faces lots of challenges. Nevertheless, with the continuous progress of characterization, separation, purification, and modification technologies, we firmly believe that exosomes will eventually be applied in the diagnosis and treatment of CCA.

## Data Availability

Not applicable.

## Abbreviations

CCA: Cholangiocarcinoma
EVs: Extracellular vesicles
HCC: Hepatocellular carcinoma
CSF: Cerebrospinal fluid
ILVs: Intraluminal vesicles
MVBs: Multivesicular bodies
ESCRT: Endosomal sorting complexes required for transport
TSG101: Tumor susceptibility gene 101
SNARE: Soluble *N*-ethylmaleimide-sensitive factor attachment protein receptor
Alix: Apoptosis-linked gene 2-interacting protein X
CME: Clathrin-mediated endocytosis
CDE: Caveolin-dependent endocytosis
TEM: Transmission electron microscopy
Cryo-EM: Cryo-electron microscopy
AFM: Atomic force microscopy
ELISA: Enzyme-linked immunosorbent assay
FCM: Flow cytometry
WB: Western blotting
HSP: Heat shock protein
MiRNA: MicroRNA
LncRNA: Long noncoding RNA
CircRNA: Circular RNA
NSCLC: Non-small cell lung cancer
AR: Androgen receptor
FOXP4: Forkhead box protein 4
EZH2: Enhancer of zeste 2 polycomb repressive complex 2 subunit
PTEN: Phosphatase and tensin homolog
CRC: Colorectal cancer
KLF2: Kruppel like factor 2
KLF4: Kruppel like factor 4
sE-cad: Soluble E-cadherin
TME: Tumor microenvironment
CAFs: Cancer-associated fibroblasts
EOC: Epithelial ovarian cancer
EMT: Epithelial-mesenchymal transition
GSK-3β: Glycogen synthase kinase 3 beta
OSCC: Oral squamous cell carcinoma
MMP9: Matrix metalloproteinase 9
CSF-1: Colony stimulating factor 1
MCP-1: Monocyte chemoattractant protein-1
TGFβ: Transforming growth factor beta
UHRF1: Ubiquitin-like with PHD and ring finger domain 1
NKs: Natural killer cells
TIM-3: T cell immunoglobulin domain and mucin domain 3
DCs: Dendritic cells
AFP: α-Fetoprotein
PD-L1: Programmed death 1 ligand
PD-1: Programmed death 1
SNHG7: Small nucleolar RNA host gene 7
LUAD: Lung adenocarcinoma
TAM: Tumor-associated macrophage
ATG5: Autophagy related 5
GPC1: Glypican-1
KEGG: Kyoto encyclopedia of genes and genomes
BMSCs: Bone marrow mesenchymal stem cells
α-SMA: Alpha-smooth muscle actin
FAP: Fibroblast activation protein alpha
IL-6: Interleukin- 6
STAT3: Signal transducer and activator of transcription 3
LY6E: Lymphocyte antigen 6 family member E
CCAC1: Cholangiocarcinoma-associated circular RNA 1
YY1: Yin Yang 1
CAMLG: Calcium modulating ligand
FZD 10: Frizzled class receptor 10
CIKs: Cytokine-induced killer cells
TNF-α: Tumor necrosis factor-α
PSC: Primary sclerosing cholangitis
UC: Ulcerative colitis
CMIP: C-Maf inducing protein
GAD1: Glutamate decarboxylase 1
NDKP1: Nucleoside diphosphate kinase 1
CDS1: CDP-diacylglycerol synthase 1
CKS1B: Cyclin-dependent kinase regulatory subunit 1
UBE2C: Ubiquitin-conjugating enzyme E2C
SERPINB1: Serine protease inhibitor B1
MALAT1: Metastasis associated lung adenocarcinoma transcript 1
Cripto-1: Teratocarcinoma-derived growth factor 1 (TDGF-1)
PHCCA: Perihilar cholangiocarcinoma
AMPN: Aminopeptidase N
VNN1: Pantetheinase
PIGR: Polymeric immunoglobulin receptor
FIBG: Fibrinogen gamma chain
A1AG1: Alpha1-acid glycoprotein 1
S100A8: S100 calcium binding protein A8
iCCA: Intrahepatic cholangiocarcinoma
pCCA: Perihilar cholangiocarcinoma
dCCA: Distal cholangiocarcinoma
IDH1/2: Isocitrate dehydrogenase1/2
KRAS: Kirsten ratsarcoma viral oncogene homolog
BAP1: BRCA1 associated protein 1
TP53: Tumor protein p53
FGFR: Fibroblast growth factor receptor
PRKACA: Protein kinase cAMP-activated catalytic subunit alpha
PRKACB: Protein kinase cAMP-activated catalytic subunit beta
ELF3: E74 like ETS transcription factor 3
AMSCs: Adipose tissue-derived mesenchymal stem cells
mTOR: Mechanistic target of rapamycin kinase
